# Dietary Collagen Hydrolysates Ameliorate Furrowed and Parched Skin Caused by Photoaging in Hairless Mice

**DOI:** 10.3390/ijms22116137

**Published:** 2021-06-07

**Authors:** Min-Kyung Kang, Dong-Yeon Kim, Hyeongjoo Oh, Soo-Il Kim, Su-Yeon Oh, Woojin Na, Sin-Hye Park, Kyungho Park, Jun-Il Kim, Ae-Hyang Kim, Young-Hee Kang

**Affiliations:** 1 Department of Food and Nutrition, Hallym University, Chuncheon 24252, Korea; mitholy@hallym.ac.kr (M.-K.K.); ehddus3290@naver.com (D.-Y.K.); ohhyeongju@gmail.com (H.O.); ky4850@naver.com (S.-I.K.); suy0411@naver.com (S.-Y.O.); nsm0729@hanmail.net (W.N.); sinhyepark@hallym.ac.kr (S.-H.P.); Kyungho.Park@hallym.ac.kr (K.P.); 2 Healthcare & Nutrition Laboratory, Amicogen Inc., Jinju 52621, Korea; jikim@amicogen.com (J.-I.K.); ahkim@amicogen.com (A.-H.K.)

**Keywords:** fish skin collagen hydrolysates, moisturizing, photoaging, skin barrier, wrinkle

## Abstract

Collagen hydrolysates have been suggested as a favorable antiaging modality in skin photoaged by persistent exposure to ultraviolet radiation (UV). The current study evaluated the beneficial effect of collagen hydrolysates (fsCH) extracted from *Pangasius hypophthalmus* fish skin on wrinkle formation and moisture preservation in dorsal skin of hairless mice challenged with UV-B. Inter-comparative experiments were conducted for anti-photoaging among fsCH, retinoic acid (RA), N-acetyl-D-glucosamine (NAG), and glycine-proline-hydroxyproline (GPH). Treating human HaCaT keratinocytes with 100−200 μg/mL fsCH reciprocally ameliorated the expression of aquaporin 3 (AQP3) and CD44 deranged by UV-B. The UV-B-induced deep furrows and skin thickening were improved in parched dorsal skin of mice supplemented with 206–412 mg/kg fsCH as well as RA and GPH. The UV-B irradiation enhanced collagen fiber loss in the dorsal dermis, which was attenuated by fsCH through enhancing procollagen conversion to collagen. The matrix metalloproteinase expression by UV-B in dorsal skin was diminished by fsCH, similar to RA and GPH, via blockade of collagen degradation. Supplementing fsCH to UV-B-irradiated mice decreased transepidermal water loss in dorsal skin with reduced AQP3 level and restored keratinocyte expression of filaggrin. The expression of hyaluronic acid synthase 2 and hyaluronidase 1 by UV-B was remarkably ameliorated with increased production of hyaluronic acid by treating fsCH to photoaged mice. Taken together, fsCH attenuated photoaging typical of deep wrinkles, epidermal thickening, and skin water loss, like NAG, RA, or GPH, through inhibiting collagen destruction and epidermal barrier impairment.

## 1. Introduction

Skin photoaging is caused by persistent exposure to ultraviolet radiation (UV) sources [1,2,3]. Clinical symptoms of photoaging include fine and coarse wrinkles, alterations in pigmentation, and histological changes of skin roughness and dryness, consequently resulting in severe atrophy, skin cancer, and melanoma [1,4]. Several mechanisms underpinning the damaging effects of acute and chronic exposure to UV radiation are related to reactive oxygen species (ROS) production and DNA damage, inflammation, immunosuppression, and extracellular matrix (ECM) remodeling and angiogenesis [2,5,6]. ROS plays a role in the upregulation of matrix-metalloproteinases (MMP) responsible for connective tissue degradation in photoaging and metastasis [1,4,7]. The damaging alterations in ECM by MMP cause skin wrinkling, angiogenesis, and tumor cell invasion, contributing to the occurrence of premature skin aging and cancer [7]. Inflammation is another important mediator in UV radiation-induced photoaging and photocarcinogenesis, in which diverse inflammatory mediators including lipid mediators and inflammatory cytokines are involved [5]. The inflammatory process stimulates production of ROS and reactive nitrogen species, leading to generation of peroxynitrites damaging DNA [5,8]. The current pharmaceutical approaches for managing the photoaging process consider the mechanisms of photoaging and cutaneous aging [3,9]. Nevertheless, prevention via sensible photoprotection remains the best current management option.

The ECM of the skin comprises various proteins such as collagens, elastin, fibronectin, and proteoglycans, where collagens are the most prevalent fiber-forming protein (77% of the fat-free dry weight of human skin) [10]. Collagens provide the elasticity and stability of connective tissues such as cartilage, bones, tendons, and ligaments as well as the skin [11]. Collagen damage in aged or photodamaged skin can be attributed to degradation by MMP released from epidermal keratinocytes and dermal fibroblasts [12]. However, possible mechanisms responsible for collagen destruction are not fully understood. Collagen has a unique right-handed triple helical structure and comprises three left-handed polyproline-like helices, each with a glycine-X-Y repeating sequence, where X and Y are often proline and hydroxyproline [13]. The tripeptide glycine-proline-hydroxyproline (GPH) is considered to be a collagen-specific sequence [14,15]. This tripeptide is initially hydrolyzed to the dipeptide proline-hydroxyproline by the intestinal epithelium-bound aminopeptidase N on the brush border membrane [14,16]. This dipeptide, a major active constituent of collagen-derived peptides, can be transported into enterocytes via the H+-coupled oligopeptide transporter [14].

**Figure 1:**
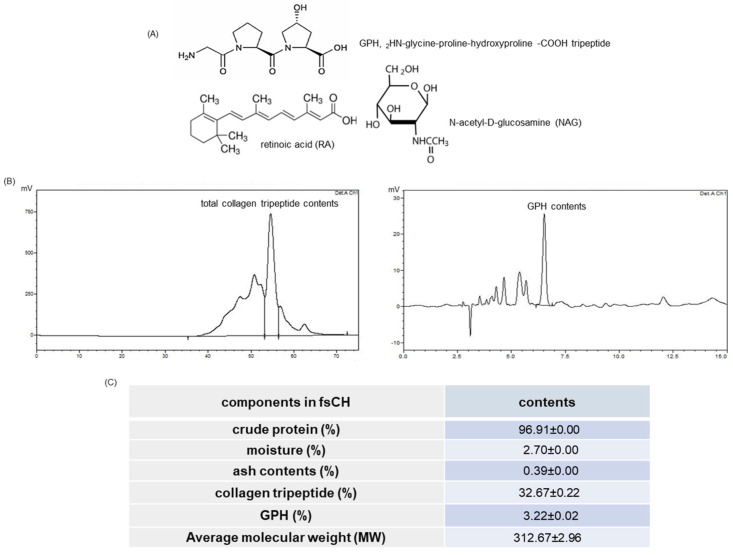
(**A**) Chemical structures of glycine-proline-hydroxyproline tripeptide (GPH), retinoic acid (RA), and N-acetyl-D-glucosamine (NAG) and the HPLC spectra (**B**) and contents (**C**) for total collagen tripeptides and GPH obtained from *Pangasius hypophthalmus* fish skin hydrolysates (fsCH), by using SuperdexT^TM^ Peptide 10/300GL column and Zorbax SB-AQ column. The peaks of collagen tripeptides and GPH were identified by the ultraviolet contour plot obtained by a photodiode-array detector at the wavelengths of 214 and 220 nm.

Several studies have reported beneficial effects of collagen hydrolysates on joint pains, wound healing, and epidermal barrier function [17,18]. In addition, the functional peptide of proline-hydroxyproline derived from dietary collagens can be transferred to the skin [14]. The intake of collagen hydrolysates improves loss of epidermal barrier function, skin elasticity, and photoaging [19,20]. Nevertheless, the mechanism(s) underlying the beneficial effects of collagen hydrolysates remains to be defined. Based on the beneficial effects of collagen hydrolysates on dry skin [19,20], the current study evaluated the skin protection of novel *Pangasius hypophthalmus* fish skin collagen hydrolysates (fsCH) containing collagen tripeptides, by examining wrinkle formation and moisture preservation in dorsal skin of hairless mice challenged with 100 mJ/cm^2^ UV-B. One report shows that collagen tripeptide supplementation improves photoaging and epidermal skin barrier in UV-B-exposed hairless mice [21]. The current experiments examined wrinkle formation, skin thickening, transepidermal water loss (TEWL), and skin hydration. Further, the skin moisturizing and anti-wrinkle effects of fsCH were investigated in keratinocytes and fibroblasts exposed to 30 mJ/cm^2^ UV-B. This study conducted inter-comparative experiments for anti-photoaging among fsCH, retinoic acid (RA), N-acetyl-D-glucosamine (NAG), and GPH (Figure 1A). A recent report demonstrates that RA and its derivatives have therapeutic potential for prevention of various dermatological disorders [22]. Topical NAG leads to an increase in skin moisturization, a decrease in skin flakiness, and the normalization of stratum corneum exfoliation [23].

## 2. Results

### 2.1. HPLC Spectra and Collagen Tripeptide Contents of fsCH

To find out how the amount of collagen tripeptides the novel fsCH contained, HPLC analyses were conducted. Figure 1B shows the contents of total collagen tripeptides and GPH calculated from the peak areas of HPLC spectra. The crude proteins formed ~97% in fsCH, and the contents of CTP and GPH were 32.67 ± 0.22% and 3.22 ± 0.02%, respectively (Figure 1C).

### 2.2. Changes in Body and Skin Weights and Liver Toxicity Following UV-B Challenge

**Figure 2:**
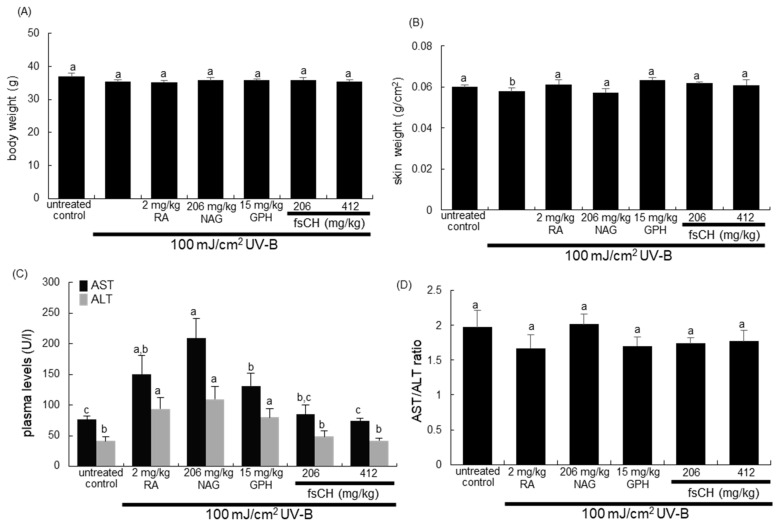
Body and skin weights (**A**,**B**), and the serum levels (**C**) and ratio (**D**) of aspartate aminotransferase (AST) and alanine aminotransferase (ALT) of SKH-1 hairless mice. SKH-1 hairless mice were exposed to UV-B three times a week while being treated with 2 mg/kg retinoic acid (RA) intraperitoneally and 206 mg/kg N-acetyl-D-glucosamine (NAG), 15 mg/kg glycine-proline-hydroxyproline tripeptide (GPH), and 206–412 mg/kg *Pangasius hypophthalmus* fish skin hydrolysates (fsCH) orally for eight weeks. The ultraviolet-B irradiation intensity increased weekly by 1 MED up to 3 MED until the eighth week and total irradiation intensity was 63 MED inflicted to mice during eight weeks. Respective values (*n* = 15) in bar graphs not sharing a small letter are different at *p* < 0.05.

The body and skin weights were not changed by UV-B challenge and all treatments of RA, NAG, GHP, and fsCH (Figure 2A,B). Unlike fsCH, the treatments of RA, NAG, and GPH increased the plasma levels of aspartate aminotransferase (AST) and alanine aminotransferase (ALT) during UV-B irradiation (Figure 2C). However, the plasma AST/ALT ratio remained unchanged in RA-, NAG-, and GPH-supplemented mice, indicating that the UV-B challenge and all treatments did not cause liver toxicity (Figure 2D).

### 2.3. Blockade of Impairment of Skin Barrier Function by UV-B Irradiation by fsCH

**Figure 3:**
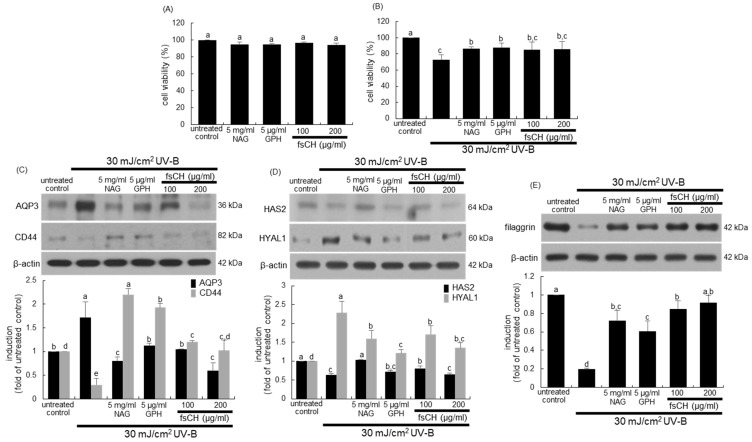
Cytotoxic responses of HaCaT keratinocytes to N-acetyl-D-glucosamine (NAG), glycine-proline-hydroxyproline tripeptide (GPH), and *Pangasius hypophthalmus* fish skin hydrolysates (fsCH) (**A**,**B**) and effects of fsCH on target protein levels in the absence and presence of 30 mJ/cm^2^ ultraviolet-B (UV-B) irradiation (**C**–**E**). Human HaCaT keratinocytes were incubated with 5 mg/mL NAG, 5 μg/mL GPH, and 100–200 μg/mL fsCH and challenged with UV-B irradiation for two days. Cell viability (mean ± SEM, *n* = 5) was measured by MTT assay and expressed as the percent cell survival compared to controls (**A**,**B**). HaCaT cell lysates were electrophoresed on 8–10% SDS-PAGE and subjected to Western blot analysis with a primary antibody against aquaporin 3 (AQP3), CD44, hyaluronic acid synthase 2 (HAS2), hyaluronidase 1 (HYAL1) and filaggrin (**C**–**E**). β-Actin antibody was used as an internal control. The bar graphs (mean ± SEM, *n* = 3) in the bottom panels represent quantitative results of the blot bands on top obtained from a densitometer. Respective values in bar graphs not sharing a small letter are different at *p* < 0.05.

To determine the appropriate treatment amounts of NAG, GPH, and fsCH, the cytotoxicity assay was performed. There was no noticeable toxicity observed in HaCaT keratinocytes treated with 5 mg/mL NAG, 5 μg/mL GPH, and 100–200 μg/mL fsCH (Figure 3A). When keratinocytes, a representative cell type in the epidermis, were exposed to 30 mJ/cm^2^ UV-B for 48 h, the cell viability was reduced by ~30% (Figure 3B). However, the treatments of NAG and GPH enhanced the viability significantly (Figure 3B). In addition, there was a considerable but not significant increase in viability of fsCH-treated keratinocytes (Figure 3B).

It was examined whether fsCH ameliorated the skin barrier function of keratinocytes impaired by UV-B irradiation. The irradiation of keratinocytes with 30 mJ/cm^2^ UV-B augmented the level of the water channel aquaporin 3 (AQP3), while the expression of the hyaluronic acid receptor CD44 was downregulated (Figure 3C). The protein expression of AQP3 by 30 mJ/cm^2^ UV-B was restored to the untreated control level in keratinocytes treated with 5 μg/mL GPH and 100 μg/mL fsCH (Figure 3C). Treating 5 mg/mL NAG and 200 μg/mL fsCH to keratinocytes further reduced the AQP3 level. On the other hand, the reduced CD44 expression was highly enhanced in all treatments with NAG, GPH, and fsCH (Figure 3C). It should be noted that the CD44 expression in keratinocytes treated with NAG and GPH increased by ~2-fold compared to that of untreated control.

Hyaluronic acid is one of the most abundant components of the cutaneous ECM and is involved in tissue homeostasis, hydration, and repair processes [24,25]. The present experiments examined the regulation of hyaluronic acid metabolism in keratinocytes during fsCH treatment. The current study evaluated expression of the hyaluronic acid-synthesizing enzyme hyaluronic acid synthase 2 (HAS2) and the hyaluronic acid-degrading enzyme hyaluronidase 1 (HYAL1) in response to fsCH treatment. The HAS2 expression was markedly suppressed by UV-B exposure of cultured HaCaT keratinocytes (Figure 3D). On the contrary, the HYAL1 expression highly increased in UV-B-exposed keratinocytes (Figure 3D). When keratinocytes were treated with 5 mg/mL NAG, 5 μg/mL GPH, and 100–200 μg/mL fsCH during UV-B irradiation, all the treatments reduced the HYAL1 expression, but the HAS2 expression was significantly enhanced only by 5 mg/mL NAG and 100 μg/mL fsCH (Figure 3D).

This study further found that fsCH influenced the expression of filaggrin, a filament-related protein that binds to keratin fibers in epithelial cells, in the presence of UV-B radiation. The filaggrin expression in keratinocytes markedly decreased due to UV-B irradiation (Figure 3E). In contrast, the supplement of keratinocytes with NAG, GPH, and fsCH restored its expression.

### 2.4. Inhibition of UV-B-Induced Collagen Loss in Dermal Fibroblasts by fsCH

**Figure 4:**
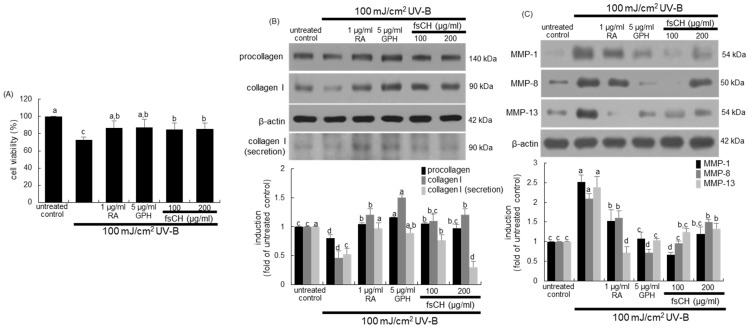
Cytotoxic responses of dermal fibroblasts to retinoic acid (RA), glycine-proline-hydroxyproline tripeptide (GPH), and *Pangasius hypophthalmus* fish skin hydrolysates (fsCH) in the absence and presence of 100 mJ/cm^2^ ultraviolet-B (UV-B) irradiation (**A**) and effects of fsCH on expression of target proteins (**B**,**C**). Human dermal fibroblasts were incubated with 1 μg/mL RA, 5 μg/mL GPH, and 100–200 μg/mL fsCH and challenged with UV-B irradiation for two days. Cell viability (mean ± SEM, *n* = 5) was measured by MTT assay and expressed as percent cell survival compare to untreated controls (**A**). Fibroblast lysates and culture media were electrophoresed on 8–10% SDS-PAGE and subjected to Western blot analysis with a primary antibody against procollagen, type 1 collagen, matrix metalloproteinase (MMP)-1, MMP-8, and MMP-13 (**B**,**C**). β-Actin antibody was used as an internal control. The bar graphs (mean ± SEM, *n* = 3) in the bottom panels represent quantitative results of the blot bands on top obtained from a densitometer. Respective values in bar graphs not sharing a small letter are different at *p* < 0.05.

When fibroblasts, a representative cell type in the dermis, were exposed to 100 mJ/cm^2^ UV-B for 48 h, ~30% of cells died (Figure 4A). Supplying 1 µg/mL RA, 5 μg/mL GPH, and 100–200 μg/mL fsCH to fibroblasts exposed to UV-B radiation ameliorated the cell viability significantly (Figure 4A).

Collagen-degrading MMP proteins are responsible for winkle formation in photoaging [26]. It was investigated that fsCH attenuated the collagen destruction induced by UV-B irradiation. As expected, the UV-B irradiation diminished the cellular levels of procollagen and type 1 collagen in dermal fibroblasts (Figure 4B). However, the reduced levels were significantly enhanced in all the treatments of fibroblasts with 1 μg/mL RA, 5 μg/mL GPH, and 100–200 μg/mL fsCH. Interestingly, supplementing RA, GPH, and fsCH to UV-B-exposed fibroblasts further promoted cellular formation of type 1 collagen (Figure 4B). In addition, the collagen secretion was enhanced in a similar manner to its formation by RA, GPH, and fsCH, as evidenced by Western blot analysis (Figure 4B). On the other hand, the UV-B irradiation augmented cellular expression of MMP-1, MMP-8, and MMP-13 in fibroblasts (Figure 4C). The cellular expression of the MMP proteins was remarkably reduced by treating 5 μg/mL GPH and 100 μg/mL fsCH to UV-B-irradiated fibroblasts (Figure 4C). In particular, the treatment of 1 μg/mL RA dampened the MMP-13 expression enhanced by UV-B radiation. However, the higher dose of fsCH was not effective in further reducing the expression of MMP-1 and MMP-8 (Figure 4C).

### 2.5. Inhibition of UV-B-Induced Wrinkle Formation by fsCH

**Figure 5:**
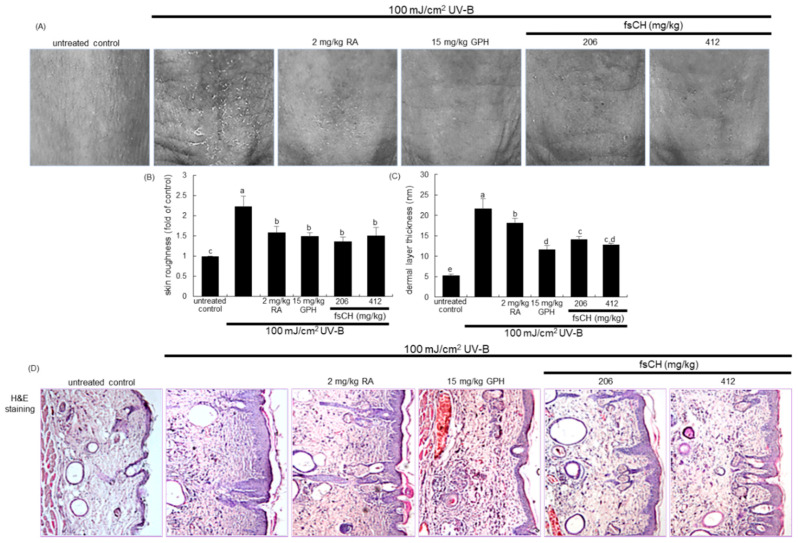
Wrinkle formation (**A**), skin roughness (**B**), dermal layer thickness (**C**), and dermal morphology (**D**) of SKH-1 hairless mouse dorsal skin. SKH-1 hairless mice were exposed to ultraviolet-B (UV-B) three times a week and treated with 2 mg/kg retinoic acid (RA) intraperitoneally and with 15 mg/kg glycine-proline-hydroxyproline tripeptide (GPH) and 206–412 mg/kg *Pangasius hypophthalmus* fish skin hydrolysates (fsCH) orally for eight weeks. Skin wrinkle images were photographed by using skin visiometer (**A**), and skin roughness and dermal layer thickness were measured from photographs by microscopic analysis (mean ± SEM, *n* = 5) (**B**,**C**). Respective values in bar graphs not sharing a small letter are different at *p* < 0.05. For the morphometric analysis of skin, dermal tissue sections were stained with H&E (**D**). Each photograph is representative of five mice.

To examine whether fsCH alleviated the wrinkle formation induced by UV-B irradiation, 206–412 mg/kg fsCH was orally administrated to male SKH-1 hairless mice exposed to 100 mJ/cm^2^ UV-B for eight weeks. Meanwhile, mice were also treated with 15 mg/kg GPH orally and 2 mg/kg RA intraperitoneally as positive controls. The UV-B irradiation notably induced the formation of coarse wrinkles in the dorsal skin (Figure 5A). However, deep furrows noticeably disappeared in dorsal skin of mice treated with RA, GPH, and fsCH. Further, the image analysis was employed in order to quantify the skin surface roughness and thickness. The skin roughness and the dermal layer thickness were highly augmented in mice irradiated with 100 mJ/cm^2^ UV-B (Figure 5B,C). When RA, GPH, and fsCH were administrated to UV-B-exposed mice, the skin roughness was reduced (Figure 5B). In addition, the treatment of mice with RA, GPH, and fsCH lessened the dermal layer thickness elevated by UV-B irradiation by different extents (Figure 5C).

This experiment attempted to visualize the skin epidermis of UV-B-irradiated hairless mice with hematoxylin–eosin (H&E) stain dye. There was a thicker dorsal epidermis observed in UV-B-exposed mice, compared with that in untreated control (Figure 5D). Consistently, increased epidermal thickness in the dorsal skin by UV-B appeared to decrease owing to supplementation of RA, GPH, and fsCH. Interestingly, collagen tripeptides such as 15 mg/kg GPH and 206–412 mg/kg fsCH were more effective in reducing dermal layer thickness than 2 mg/kg RA (Figure 5C,D).

### 2.6. Inhibition of UV-B-Induced Collagen Loss by fsCH

**Figure 6:**
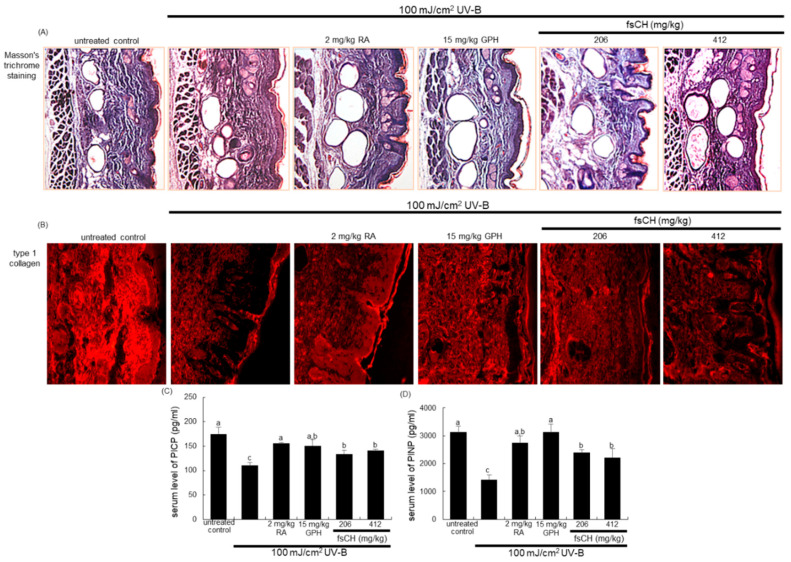
Effects of oral administration of *Pangasius hypophthalmus* fish skin hydrolysates (fsCH) on ultraviolet-B (UV-B)-induced collagen loss of hairless mouse dorsal skin. SKH-1 hairless mice were exposed to UV-B radiation three times a week and treated with 2 mg/kg retinoic acid (RA) intraperitoneally and with 15 mg/kg glycine-proline-hydroxyproline tripeptide (GPH) and 206–412 mg/kg fsCH orally for eight weeks. Masson’s trichrome staining was performed with mouse dermal skin for collagen fibers stained in blue (**A**). For the measurement of dorsal skin tissue level of type 1 collagen in UV-B-irradiated SKH-1 hairless mice, immunohistochemical staining was conducted with an antibody against type 1 collagen (**B**). Each photograph is representative of four animals. Serum levels of procollagen type 1 carboxy-terminal propeptide (PICP) and procollagen type 1 amino-terminal propeptide (PINP) were measured by using ELISA kits (**C**,**D**). Values in bar graphs (mean ± SEM, *n* = 3) not sharing a small letter are different at *p* < 0.05.

This experiment examined whether 206–412 mg/kg fsCH promoted collagen synthesis and conversely inhibited its degradation in UV-B exposed mouse dorsal skin, as assessed by Masson trichrome staining. UV-B-irradiated SKH-1 hairless mice showed a significant decrease in the abundance and density of blue-colored collagen fibers in the dermis, compared with that in untreated control mice (Figure 6A). However, the treatment with 2 mg/kg RA, 15 mg/kg GPH, and 206 mg/kg fsCH inhibited the UV-B irradiation-induced loss of collagen fibers. In addition, immunohistochemical staining revealed that UV-B irradiation diminished dermal tissue level of Cy3-stained type 1 collagen (Figure 6B). When RA, GPH, and 206 mg/kg fsCH were administrated to mice challenged to UV-B, the production of type 1 collagen was highly enhanced in mouse dorsal skin (Figure 6B). These results indicate that GPH and fsCH as well as the positive control RA can attenuate UV-B irradiation-induced skin thickening and collagen fiber loss.

The current study further investigated favorable effects of fsCH on collagen formation in mice exposed to UV-B. The procollagen propeptides of procollagen type 1 carboxy-terminal propeptide (PICP) and procollagen type 1 amino-terminal propeptide (PINP) are released in equimolar concentrations into the circulation during the extracellular conversion of procollagen to collagen, identified as an indicator of type 1 collagen synthesis in skin recovery [27]. UV-B irradiation reduced serum levels of PICP and PINP in mice, as evidenced by enzyme-linked immunosorbent assay (ELISA, Figure 6C,D). The treatment with RA and GPH highly elevated the serum levels of these propeptides deterred by UV-B. In addition, supplying fsCH orally to mice substantially increased serum levels of PICP and PINP (Figure 6C,D). Thus, similar to RA, fsCH may ameliorate skin collagen synthesis damaged by chronic treatment of UV-B.

### 2.7. Blockade of UV-B Expression of MMP Proteins by fsCH

**Figure 7:**
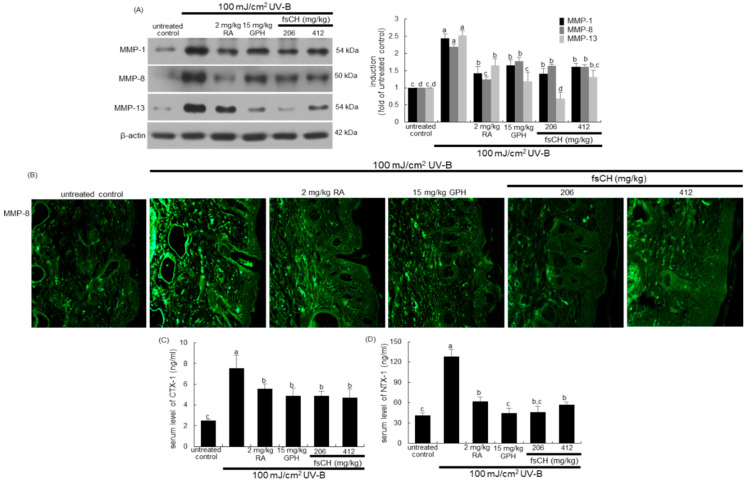
Effects of *Pangasius hypophthalmus* fish skin hydrolysates (fsCH) on dermal expression of matrix metalloproteinases (MMP) and collagen degradation in ultraviolet-B (UV-B)-irradiated SKH-1 hairless mice. SKH-1 hairless mice were exposed to UV-B radiation three times a week and treated with 2 mg/kg retinoic acid (RA) intraperitoneally and with 15 mg/kg glycine-proline-hydroxyproline tripeptide (GPH) and 206–412 mg/kg fsCH orally for eight weeks. Skin tissue extracts were electrophoresed on 8–12% SDS-PAGE and subjected to Western blot analysis with a primary antibody against MMP-1, MMP-8, and MMP-13 (**A**). β-Actin antibody was used as an internal control. The bar graphs (mean ± SEM, *n* = 3) in the right panels represent quantitative results of the blot bands on the left obtained from a densitometer. The skin tissue MMP-8 expression was immunohistochemically identified by FITC-green staining (**B**). Serum levels of carboxy-terminal telopeptide of type 1 collagen (CTX-1) and amino-terminal telopeptide of type 1 collagen (NTX-1) were measured by using ELISA kits ((**C**,**D**), *n* = 7). Respective values in bar graphs not sharing a small letter are different at *p* < 0.05.

It was found that fsCH inhibited activation of collagen-degrading enzymes of MMP-1, MMP-8, and MMP-13 in mouse dorsal skin by UV-B irradiation. Western blot data show that UV-B irradiation induced these MMP proteins in dorsal skin tissues, which was diminished by the treatment of mice with RA, GPH, and fsCH (Figure 7A). Interestingly, the individual treatment inhibited the respective MMP proteins in their own ways. On the other hand, there was heavy FITC-green staining of MMP-8 detected in mouse dorsal skin, indicating that this irradiation enhanced the MMP-8 expression (Figure 7B). In contrast, the treatment with RA, GPH, and fsCH attenuated the MMP-8 expression. Thus, the treatments of GPH and fsCH may block collagen breakdown in UV-B exposed skin tissues.

This experiment examined whether fsCH influenced serum levels of carboxy-terminal telopeptide of type 1 collagen (CTX-1) and amino-terminal telopeptide of type 1 collagen (NTX-1), both released during collagen degradation. The release of CTX-1 and NTX-1 into the circulation was enhanced in UV-B-irradiated mice (Figure 7C,D). Increased release of both biomarkers responsible for collagen degradation was reduced by all the treatments with RA, GPH, and fsCH in a similar manner.

### 2.8. Preservation of Dorsal Skin Hydration by fsCH in UV-B-Irradiated Mice

**Figure 8:**
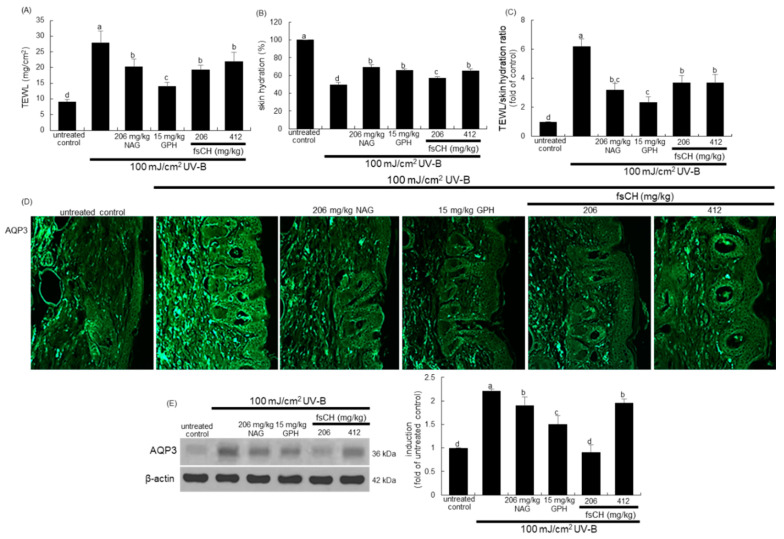
Effects of *Pangasius hypophthalmus* fish skin hydrolysates (fsCH) on transepidermal water loss (TEWL), skin hydration, and aquaporin 3 (AQP3) level in ultraviolet-B (UV-B) radiation-exposed mice. SKH-1 hairless mice were exposed to UV-B radiation three times a week and treated with 206 mg/kg N-acetyl-D-glucosamine (NAG), 15 mg/kg glycine-proline-hydroxyproline tripeptide (GPH), and 206–412 mg/kg fsCH orally for eight weeks. TEWL (**A**), skin hydration (**B**), and TEWL/skin hydration ratio (**C**) were measured and calculated. The AQP3 level of skin tissues was immunohistochemically confirmed by FITC-green staining (**D**). Skin tissue extracts were electrophoresed on 10% SDS-PAGE and subjected to Western blot analysis with a primary antibody against AQP3 (**E**). β-Actin antibody was used as an internal control. The bar graph (mean ± SEM, *n* = 3) in the right panel represents the quantitative results of blot band on the left obtained from a densitometer. Respective values in bar graphs not sharing a small letter are different at *p* < 0.05.

The present study investigated that fsCH preserved water retention in dorsal skin damaged in UV-B-irradiated mice. Transepidermal water loss (TEWL) is the amount of water that passively evaporates through skin to the external environment and is used to characterize skin barrier function [28]. The UV-B radiation resulted in an increase in TEWL and a reciprocal reduction of epidermal hydration in mice (Figure 8A,B). The values of TEWL and skin hydration were much lower in mice treated with 206 mg/kg NAG, 15 mg/kg GPH, and 206–412 mg/kg fsCH, compared with that in mice exposed to UV-B-alone. In addition, the GPH treatment showed the lowest TEWL/skin hydration ratio, indicating that GPH was most effective in preserving skin moisturizing (Figure 8C).

To confirm that fsCH was effective in moisturizing dorsal skin impaired by UV-B radiation, the epidermal moisturizing function was examined. The immunohistochemical data show that the expression of green FITC-conjugated AQP3 was highly prompted in UV-B-challenged mice (Figure 8D). The FITC-green staining intensity of AQP3 in dorsal skin was attenuated by oral treatment of NAG, GPH, and fsCH to UV-B-exposed mice. Consistent with immunohistochemical data, Western blot data show that fsCH mitigated the expression level of the water channel AQP3, possibly blocking water evaporation from the epidermis (Figure 8E).

### 2.9. Maintenance of Skin Barrier Function by fsCH in UV-B-Exposed Mice

**Figure 9:**
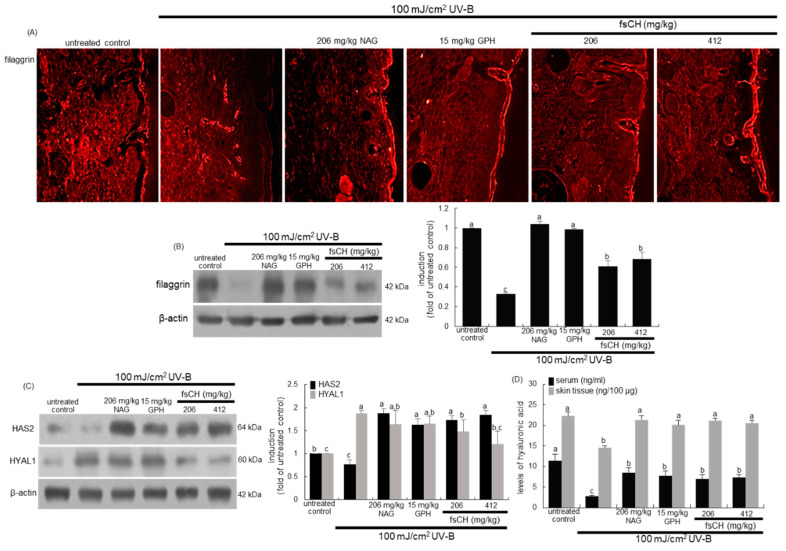
Effects of *Pangasius hypophthalmus* fish skin hydrolysates (fsCH) on skin tissue levels of filaggrin, hyaluronic acid synthase 2 (HAS2) and hyaluronidase 1 (HYAL1), and serum and skin tissue levels of hyaluronic acid of UV-B-irradiated SKH-1 hairless mice. SKH-1 hairless mice were exposed to ultraviolet-B (UV-B) radiation three times a week and treated with 206 mg/kg N-acetyl-D-glucosamine (NAG), 15 mg/kg glycine-proline-hydroxyproline tripeptide (GPH), and 206–412 mg/kg fsCH orally for eight weeks. The filaggrin localization was visualized by a Cy3-red staining (**A**). Skin tissue extracts were electrophoresed on 8–12% SDS-PAGE and subjected to Western blot analysis with a primary antibody against filaggrin, HAS2, and HYAL1 (**B**,**C**). β-Actin antibody was used as an internal control. The bar graphs (mean ± SEM, *n* = 3) in the right panels represent the quantitative results of the blot bands on the left obtained from a densitometer. Serum and skin tissue levels of hyaluronic acid were measured by using ELISA kits (**D**). Respective values in bar graphs not sharing a small letter are different at *p* < 0.05.

It was further found that fsCH ameliorated the impairment of epidermal permeability barrier function of mouse dorsal skin by UV-B radiation. There was weak staining of red Cy3-conjugated filaggrin in the epidermis exposed to UV-B (Figure 9A). On the contrary, the treatments of 206 mg/kg NAG, 15 mg/kg GPH, and 206–412 mg/kg fsCH restored the decreased filaggrin expression. Consistently, the epidermal expression of filaggrin was dampened in animals (Figure 9B). However, supplementing NAG and GPH orally to UV-B-exposed mice recovered epidermal filaggrin near-completely to its level of untreated control mice. In addition, fsCH substantially enhanced the filaggrin expression, minimizing water loss in the epidermis (Figure 9B).

Figure 9C shows that UV-B radiation influenced the protein levels of HAS2 and HYAL1 in the skin of hairless mice. Oral supplementation of 206 mg/kg NAG, 15 mg/kg GPH, and 206–412 mg/kg fsCH remarkably enhanced HAS2 expression impaired by UV-B irradiation (Figure 9C). On the contrary, the HYAL1 protein levels increased in UV-B-irradiated mouse skin, which was inhibited by supplying fsCH to mice (Figure 9C). It should be noted that the in vivo mouse data for HAS2 and HYAL1 were not consistent with their in vitro data with cultured keratinocytes. Nevertheless, it was assumed that fsCH contributed to the moisturizing effect on UV-B-damaged dorsal skin. Furthermore, 100 mJ/cm^2^ UV-B dampened the hyaluronic acid levels in the serum and dermal tissues, as assessed by ELISA (Figure 9D). However, the treatment of mice with NAG, GPH, and fsCH enhanced the serum level of hyaluronic acid reduced by UV-B radiation. In addition, the dermal tissue level of hyaluronic acid was almost restored by all the treatment to the basal level of untreated control mice (Figure 9D).

## 3. Discussion

**Figure 10:**
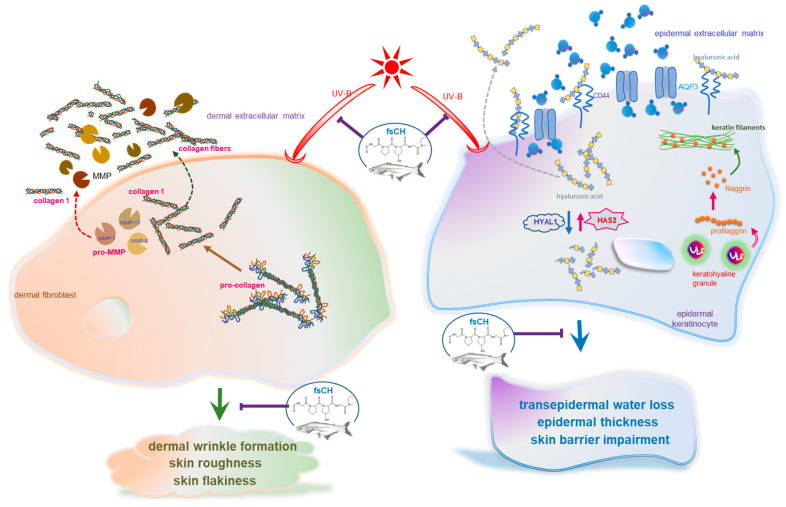
Schematic diagram showing the beneficial effects of *Pangasius hypophthalmus* fish skin hydrolysates (fsCH) on UV-B radiation-damaged skin of SKH-1 hairless mice. As depicted, fsCH attenuated dermal wrinkle formation in fibroblasts and epidermal water loss in keratinocytes. The symbol ⟘ indicates sites of inhibition manifested by fsCH and the symbol → indicates activation. UV-B, ultraviolet-B; MMP, matrix metalloproteinases; AQP3, aquaporin 3; HAS2, hyaluronic acid synthase 2; HYAL1, hyaluronidase 1.

Eight major findings were extracted from the present study employing fsCH. (1) There was notable improvement in viability by nontoxic 100–200 μg/mL fsCH in UV-B-irradiated keratinocytes and dermal fibroblasts. (2) Treating keratinocytes with fsCH inversely modulated the UV-B-elevated AQP3 expression and reduced CD44 expression. (3) The UV-B-induced deep furrows, coarse wrinkles, and skin thickening noticeably disappeared in dorsal skins of mice supplemented with 206–412 mg/kg fsCH as well as RA and GPH, accompanying reduced skin roughness and dermal layer thickness. (4) The UV-B irradiation reduced the type 1 collagen production of dermal fibroblasts and enhanced collagen fiber loss in the mouse dorsal dermis, which was remarkably attenuated in fsCH-treated fibroblasts and mice through increasing conversion of procollagen to collagen. (5) The elevated expression of MMP-1, MMP-8, and MMP-13 by UV-B exposure of fibroblasts and dorsal skin was diminished by fsCH, similar to RA and GPH, with concurrent reduction of collagen degradation. (6) Supplementing fsCH to mice decreased TEWL with increased skin hydration evoked by UV-B radiation through inhibiting the expression of AQP3 in dorsal skin. (7) The supplementation of keratinocytes and mice with fsCH restored the expression of filaggrin dampened by UV-B irradiation, minimizing water loss in the epidermis. (8) Although the reciprocal expression of HAS2 and HYAL1 by UV-B radiation was minimally improved in fsCH-treated cultured keratinocytes, their expression was remarkably ameliorated with increased production of hyaluronic acid by treating fsCH to photoaged mice. Taken together, fsCH attenuated deep wrinkles, epidermal thickening, and parched skin typical of photoaging, similar to NAG, RA, or GPH, through inhibiting dermal collagen destruction and impairment of epidermal permeability barrier function (Figure 10).

Skin photoaging is generally characterized by wrinkle formation and loss of skin tone and resilience, consequently leading to development of severe atrophy, skin cancer, and melanoma [1,4]. Photoaged skin displays noticeable loss and alterations in structural components, ECM, and interstitial collagens of the connective tissues in the deep dermis [4,6]. This experiment showed that UV-B enhanced skin roughness and dermal layer thickness, resulting in deep furrows and parched and thickened skin. Although the photoaging mechanism remains unclear, ambient UV-irradiation generates ROS that leads to photooxidative damage of the skin, which results in biochemical and clinical characteristics of photoaging [1,3,4,6]. In addition, prolonged inflammation is a detrimental factor responsible for photoaging due to repetitive UV-B irradiation [29]. The current study did not examine oxidative stress and inflammation in UV-B-irradiated fibroblasts and keratinocytes. However, the UV-B irradiation induced the expression of matrix-degrading metalloproteases of MMP-1, MMP-8, and MMP-13 in fibroblasts. ROS stimulates the inflammatory process in the skin, which further activates the transcription of various matrixes degrading metalloproteases, leading to abnormal matrix degradation and accumulation of non-functional matrix components [5,29]. Thus, it can be assumed that ROS and inflammation cause oxidative damage to activate cellular signaling for collagen degradation in the dermal compartments, contributing to skin photoaging. Collagen destruction in photo-damaged skin can be attributed to degradation by MMP released from epidermal keratinocytes and dermal fibroblasts [12].

Skin hydration is crucial for the normal functioning of the skin outer layer stratum corneum [25,30]. Increased TEWL results in visible appearance of dry and scaly skin due to abnormal skin desquamation [25]. Hyaluronic acid, a component abundant in the epidermis, is important for maintaining normal structure stratum corneum and epidermal barrier function. The UV-B irradiation reduced skin tissue and serum levels of hyaluronic acid. Consistently, the expression of HAS2 and CD44 declined, and oppositely the HYAL1 expression increased in chronic UV-B-exposed keratinocytes of the epidermis. One report shows that chronically irradiated UV-B causes marked loss of hyaluronic acid in fibroblasts from the papillary dermis and downregulation of HAS enzyme expression [31]. Unlike the present results with keratinocytes, HYAL proteins and CD44 are unchanged in fibroblasts exposed to chronic UV-B [31]. There is a complex temporal and spatial regulation of hyaluronic acid metabolism in skin fibroblasts and keratinocytes in response to acute UVB irradiation [32]. Accordingly, chronic UV-B irradiation results in loss of hyaluronic acid from the epidermis, thereby contributing to the quiescent phenotype of epidermal keratinocytes. The water- and glycerol-transporting protein AQP3 in the viable epidermis is involved in the mechanisms responsible for various keratinocyte functions including proliferation, hydration, water retention, and barrier function [25,30,33]. Various abnormalities in this channel are observed in several skin diseases such as psoriasis and skin cancers [33]. This study found that AQP3 was induced in keratinocytes and dorsal skin tissues exposed to UV-B radiation, consequently contributing to increase in TEWL, loss of keratinocytes and parched skin.

Several strategies have been suggested to prevent photodamage caused by aberrant reactions initiated by UV [29]. Since ROS and inflammation contribute to skin photoaging, treatments with anti-inflammatory compounds and antioxidants represent promising options to prevent skin damage [4,5]. Blocking depletion of endogenous antioxidants may be another management option [4]. Retinoids inhibiting ECM damage and MMP activity in the dermal compartments may prevent photoaging [34]. RA and its derivatives have beneficial effects in various dermatological disorders [22]. The current study employed RA as a positive control to inter-compare the formation of fiber-forming collagens in dermal fibroblasts and dorsal skin exposed to UV-B. On the other hand, collagen comprises three left-handed polyproline-like helices, frequently with a GPH-repeating sequence [13], which may be a collagen-specific sequence [14,15]. The amounts of total tripeptides and GPH were 32.67 ± 0.22% and 3.22 ± 0.02%, respectively, among ~97% crude proteins in fsCH. Thus, commercially available GPH was introduced in order to confirm that it was biologically active indicator compound in fsCH. Although GPH functions as a chemotactic stimulus for dermal fibroblasts [35], there is still a lack of functional studies dealing with its skin health. The current approach with GPH-containing fsCH has managed the mechanisms of photoaging and sensible photoprotection with potential antioxidant and anti-inflammation. Similar to RA, oral treatment with GPH-containing fsCH suppressed wrinkle formation and epidermal thickening in dorsal skin, along with accumulation of dermal collagen fibers. All the treatments with fsCH, GPH-alone, and RA abrogated UV-B-induced collagen destruction in fibroblasts through enhancing the conversion of procollagen to collagen and reducing ECM degradation by MMP proteins of fibroblasts. It has been shown that the functional peptides originated from dietary collagen supplements are transported to the skin [14]. Nevertheless, the mechanism(s) underlying the beneficial effects of collagen hydrolysates on skin elasticity is still undefined.

Several studies have shown the positive effects of collagen hydrolysates on epidermal barrier function and dermal wound healing [17,18,19,20]. Supplementation of collagen tripeptides improved impairment of epidermal skin barrier in UV-B-exposed hairless mice [22]. In this study, dietary supplementation with fsCH to prevent photodamage focused on improving skin barrier integrity and function as a moisturizer similar to NAG, a precursor to hyaluronic acid. NAG is recognized as a beneficial player in optimal ECM structure and hydration of the epidermis and the dermis [23,36]. An inverse ratio of TEWL, a sensitive indicator of skin irritation and hydration, is seen in atopic dermatitis and disorders of keratinization [37,38]. This study found that oral administration of fsCH containing tripeptides containing GPH displayed an inverse ratio of decreased TEWL and increased hydration in UV-B-exposed dorsal skin. The fsCH treatment reduced UV-B-induced AQP3 expression in keratinocytes, which can be stratified into dietary prevention of photodamage similar to topical NAG. This proactive dietary approach mandated the expression of HAS2, HYAL1, and CD44 in UV-B-irradiated dorsal skin, leading to marked dermal hyaluronic acid synthesis and transport. A study shows that collagen fragments suppress hyaluronic acid synthesis in skin fibroblasts in response to UV-B [39]. The manipulation of the expression of HAS2 and HYAL1 by fsCH was minimal in keratinocytes, as compared with dorsal skin. Repairing collagen destruction would favorably influence the expression of HAS2 and HYAL1 in fibroblasts irradiated to UV-B. Since filaggrin binds to and condenses the keratin cytoskeleton as a skin barrier, loss of filaggrin leads to a poorly formed stratum corneum (ichthyosis) and is also prone to water loss (xerosis) [40]. The treatments of fsCH and GPH showed irritancy potential, reversing UV-impaired skin barrier function by enhancing the protein expression of filaggrin, which compared favorably with NAG.

## 4. Materials and Methods

### 4.1. Materials

Dulbecco’s modified Eagle’s media and culture reagents were purchased from Sigma-Aldrich Chemical (St. Louis, MO, USA), as were all other reagents, unless specifically stated elsewhere. Fetal bovine serum (FBS), trypsin-ethylenediaminetetraacetic acid, and penicillin–streptomycin were purchased from Lonza (Walkersvillle, MD, USA). Rabbit polyclonal CD44 antibody was purchased from Cell Signaling Technology (Beverly, MA, USA). Rabbit polyclonal filaggrin antibody was obtained from Enzo Life Sciences (Farmingdale, NY, USA). Rabbit polyclonal procollagen antibody, and mouse monoclonal antibodies of type 1 collagen, MMP-1, MMP-8, MMP-13, AQP-3, HAS2, and HYAL1 were supplied by Santa Cruz Biotechnology (Santa Cruz, CA, USA). Mouse monoclonal β-actin antibody was provided by Sigma-Aldrich Chemical. Horseradish peroxidase (HRP)-conjugated goat anti-rabbit IgG, goat anti-mouse and donkey anti-goat IgG were purchased from Jackson ImmumnoReserch Laboratories (West Grove, PA, USA). GPH standard was obtained from Bachem (Bubendorf, Switzerland), while RA and NAG were purchased from Sigma-Aldrich Chemical.

### 4.2. Preparation of fsCH and HPLC Analysis

The current study employed the fsCH provided by Amicogen Biopharm Co. Ltd. (Sungnam, Gyeonggi-do, Korea). The fsCH was extracted from the skin of *Pangasius hypophthalmus* that was gelatinized in distilled water at 90–100 °C for 7 h and digested using collagenase from non-pathogenic bacteria of the *Bacillus* species. The resulted extracts were filtered with a 0.4 µm filter press and a 0.45 µm membrane filter to remove impurities, spray-dried and used as a sample.

The fsCH was analyzed by using HPLC system with SuperdexT^TM^ Peptide 10/300GL column from Cytiva (Marlborough, MA, USA) and Zorbax SB-AQ column (4.6 × 250 mm, 5 μm) from Agilent (Santa Clara, CA, USA), respectively. Total collagen tripeptides were determined by run at a flow rate of 0.3 mL/min for 60 min with 10 mM Tris buffer (pH 7.5) containing 0.15 M NaCl and 5 mM CaCl_2_. Elution was monitored at λ = 214 nm. GPH was determined by run at a flow rate of 1.0 mL/min with 0.1% trifluoroacetic acid, and elution was monitored at λ = 220 nm. The contents of total collagen tripeptides were calculated by using the area of 25–60 min, in which collagen tripeptides were detected. In the case of GPH, a standard curve was created using the H-Glycine-Proline-Hydroxyproline-OH standard, and then calculated by the following calculation formula: GPH content (mg/g) = C × V × F/W (where C is the standard GPH concentration (mg/mL), V is the standard volume, F is the dilution factor (10), and W is the sample weight (g)). Indeed, collagens are degraded randomly into bioactive peptides such as GPH in the gastrointestinal tract [Error!Referencesourcenotfound.].

The components of fsCH were analyzed for the method registered by the Ministry of Food and Drug Safety (Sejong, Korea). Crude proteins were analyzed by using the semimicro-Kjedahl method, and the nitrogen coefficient of fish gelatin was set to 5.56. Moisture contents were measured by using an OHAUS MB23 Moisture Analyzers, and fsCH ash contents were measured with dry ash procedures at a high temperature of 600 °C.

fsCH was dissolved in dimethyl sulfoxide (DMSO) for live culture with cells; the final culture concentration of DMSO was <0.5%.

### 4.3. Animals and UV-B Radiation

Male SKH-1 hairless mice (4 weeks old) were obtained from Charles River Laboratory (Wilmington, MA, USA). Mice were kept on a 12 h light/12 h dark cycle at 23 ± 1 °C with 50 ± 10% relative humidity under specific pathogen-free conditions, and they were fed a standard pellet laboratory chow diet obtained from Cargill Agri Purina (Sungnam, Gyeonggi-do Korea) supplied by the animal facility of Hallym University. The animals were allowed to acclimatize for a week before beginning the experiments.

Mice were divided into seven groups (*n* = 15 per group). The first group of mice was not irradiated with UV-B as untreated controls. The other mice were exposed to UV-B (λmax = 312 nm, no UV-A and UV-C emission) three times a week. The irradiation intensity increased weekly by 1 minimal erythema dose (MED, equal to 100 mJ/cm^2^) up to 3 MED, and then lasted at 3 MED from the 3rd week to the 8th week with no deadly injury [41]. Accordingly, total irradiation intensity inflicted to mice for 8 weeks was 63 MED, and about 7 min was required to reach 1 MED. The dorsal skin surface of these animals was irradiated with VL-206. BL UV-B lamps were purchased from Vilber Lourmet (Lamirault, Collégien, France). Among the UV-B-exposed animals, one positive group was intraperitoneally injected with 2 mg/kg RA and the other groups were orally treated with 206 mg/kg NAG, 15 mg/kg GPH, and 206–412 mg/kg fsCH for 8 weeks, based on previous reports [42,43,44]. The serum levels of AST and ALT were measured for the detection of liver toxicity of animals by using FUJI DRI-CHEM NX500i Automated Clinical Chemistry Analyzer (Tokyo, Japan).

All animal experiments were approved by the Committee on Animal Experimentation of Hallym University and performed in compliance with the Hallym University’s Guidelines for the Care and Use of Laboratory Animals (Hallym 2019-53, 1 November 2019).

### 4.4. Cell Culture and UV-B Irradiation

Human dermal fibroblasts were obtained from Clonetics (San Diego, CA, USA) and the human keratinocyte HaCaT cell line was obtained from the American Type Culture Collection (Manassas, VA, USA). Human dermal fibroblasts and the HaCaT keratinocyte cell line were cultured in Dulbecco’s modified Eagle’s media containing 10% FBS, 2 mM glutamine, 100 U/mL penicillin, and 100 μg/mL streptomycin at 37 °C in a humidified atmosphere of 5% CO_2_ in air. Fibroblasts were plated at 90–95% confluence, and keratinocytes were plated at 70–80% in all experiments. The UV-B light source was provided. Keratinocytes and fibroblasts were pre-treated with 1 μg/mL RA, 5 mg/mL NAG, 5 μg/mL GPH, or 100–200 μg/mL fsCH and exposed to 30 mJ/cm^2^ (keratinocytes) or 100 mJ/cm^2^ (fibroblasts) UV-B radiation for 48 h.

The cytotoxicity of 1 μg/mL RA, 5 mg/mL NAG, 5 μg/mL GPH, and 100–200 μg/mL fsCH was determined after 24 h culture of keratinocytes and fibroblasts by using the MTT (3-(4,5- dimethylthiazol-yl)-diphenyl tetrazolium bromide, Duchefa Biochemie, Haarlem, The Netherlands) assay. Briefly, cells were maintained in a fresh medium including 1 mg/mL MTT at 37 °C for 3 h. Gentle shaking was conducted to dissolve purple formazan product in 0.5 mL isopropanol, and the absorbance of formazan was determined at λ = 570 nm using a Bio-Rad Model 550 microplate reader (Hercules, CA, USA).

### 4.5. Western Blot Analysis

Western blot analysis was conducted with whole cell lysates and culture media prepared from human keratinocytes and dermal fibroblasts (3.5 × 10^5^ cells/well) and skin tissue extracts. Whole cell lysates, culture media, and mouse skin tissue extracts were prepared in a lysis buffer containing 1 M β-glycerophosphate, 1% β-mercaptoethanol, 0.5 M NaF, 0.1 M Na_3_VO_4_, and protease inhibitor cocktail. Cell lysates and skin tissue extracts containing equal amounts of proteins and equal volume of culture media were electrophoresed on 8–12% SDS-PAGE and transferred onto a nitrocellulose membrane. Nonspecific binding was blocked with 5% skim milk for 3 h. The membrane was incubated overnight at 4 °C with each primary antibody of target proteins, including MMP proteins, type 1 collagen, AQP-3, HAS2, and HYAL1, and thoroughly washed in a Tris-buffered saline-Tween 20 (TBS-T) for 10 min. The membrane was then incubated for 1 h with a secondary antibody of goat anti-rabbit IgG, goat anti-mouse IgG and rabbit anti-goat IgG conjugated to HRP. Each target protein level was determined by using immobilon western chemiluminescent HRP substrate from Millipore Corporation (Billerica, MA, USA) and Agfa X-ray film (Mortsel, Belgium). Incubation with mouse monoclonal β-actin antibody (Sigma-Aldrich Chemical) was also performed for comparative controls.

### 4.6. In Vivo Skin Evaluations

TEWL, an indicator of intact skin barrier function, and dorsal skin hydration were assessed by using Cutometer MPA-580 from Courage & Khazaka (Kőln, Germany) before and after each treatment. The surface changes of dorsal skin were recorded by photography.

### 4.7. Hematoxylin–Eosin Staining for Histological Observation

For the histological analyses of mouse skin, dorsal skin tissues were fixed in 4% paraformaldehyde. The paraffin-embedded specimens were sectioned at 10 μm thickness and deparaffinized. Tissue sections were exposed to H&E stain for 2 min, thoroughly washed with tab water while staining, and quickly dehydrated in 95% absolute alcohol. The H&E-stained tissue sections were examined using a Carl Zeiss optical microscope Axioimager system equipped for fluorescence illumination (Göttingen, Germany). Five images (400×) were taken from each tissue section.

### 4.8. Masson Trichrome Staining

Paraffin-embedded skin tissues were sectioned at 10 μm thickness, de-paraffinized, hydrated with deionized water, and stained with Masson trichrome for the histological visualization of collagen fibers. Briefly, a staining series was done with hematoxylin, Biebrich scarlet acid Fucshin, phosphotungstic/phosphomolybdic acid solution, and aniline blue solution. Each step was followed by carefully rinsing in deionized water. Each slide was de-hydrated with alcohol and mounted for the histological visualization. The stained tissue sections were examined using an Optical microscope Axiomager, and five images (400×) were taken for each section

### 4.9. Immunohistochemical Staining

For the immunohistochemical analysis, paraffin-embedded mouse skin tissue sections (10 μm thickness) were employed. The tissue sections were placed on glass slides, de-paraffinated, and hydrated with xylene and graded alcohol. The sections were pre-incubated in a boiled sodium citrate buffer (10 mM sodium citrate, 0.05% Tween 20, pH 6.0) for antigen retrieval. Specific primary antibody against type 1 collagen, MMP-8, AQP3, and filaggrin was incubated with the tissue sections overnight. For the visualization of type 1 collagen and filaggrin, the tissue section was stained with red Cy3-conjugated anti-mouse IgG. For the measurement of MMP-8 and AQP3, the sections were visualized with green FITC-conjugated anti-mouse IgG. The stained tissue sections were examined using an optical Axiomager microscope system, and five images (400×) were taken for each section.

### 4.10. ELISA

Serum levels of PICP, PINP, CTX-1, and NTX-1 and serum and tissue levels of hyaluronic acid were measured using ELISA kits from Novus Biologicals (Centennial, CO, USA), according to the manufacturer’s instruction.

### 4.11. Data Analysis

The data are presented as mean ± SEM. Statistical analyses were conducted using the Statistical Analysis Software package, version 6.12 (SAS Institute, Cary, NC, USA). Significance was determined by one-way ANOVA, followed by Duncan’s multiple-range test for multiple comparisons. Differences were considered significant at *p* < 0.05.

## 5. Conclusions

The current study demonstrated that fsCH abrogated UV-B-induced skin photoaging with deep wrinkles and epidermal thickening and parched skin. This inter-comparative study employed NAG, RA, or GPH as positive controls for comparison of photodamage with fsCH. The treatment with fsCH inhibited UV-B irradiation-induced collagen destruction of dermal fibroblasts through increasing conversion of type 1 collagen from procollagen synthesis and reducing collagen degradation by MMP proteins, similar to how RA and GPH inhibited wrinkle formation. In addition, the supplementation of fsCH to mice attenuated impairment of epidermal permeability barrier function, leading to epidermal water loss due to UV-B irradiation to dorsal skin. Supplying fsCH to keratinocytes reduced the expression of AQP3 and HYAL1 enhanced by UV-B radiation, while the UV-B-induced expression of HAS2 and filaggrin was diminished by fsCH. Further, this reciprocal modification in expression of AQP3, HYAL1, and HAS2 was applied to the epidermis of mice treated with fsCH. The mechanistic actions of fsCH underlying photoaging-induced skin wrinkle formation and water loss should be defined. Although fsCH may be effective in inhibiting photoaging-induced wrinkle formation and epidermal thickness and enhancing skin moisturizing, human clinical studies are required to confirm the in vivo potential of fsCH.

## Data Availability

All the data presented in this study are included in the article.

## Abbreviations

AQP3, aquaporin 3; CTX-1, carboxy-terminal telopeptide of type 1 collagen; ECM, extracellular matrix; fsCH, *Pangasius hypophthalmus* fish skin collagen hydrolysate; GPH, glycine-proline-hydroxyproline; HAS2, hyaluronic acid synthase 2; HYAL1, hyaluronidase 1; MMP, matrix metalloproteinases; NAG, N-acetyl-D-glucosamine; NTX-1; amino-terminal telopeptide of type 1 collagen; PICP, procollagen type 1 carboxy-terminal propeptide; PINP, procollagen type 1 amino-terminal propeptide; RA, retinoic acid; ROS, reactive oxygen species; TEWL, transepidermal water loss; UV, ultraviolet.
